# Human Neutrophils Produce Antifungal Extracellular Vesicles against Aspergillus fumigatus

**DOI:** 10.1128/mBio.00596-20

**Published:** 2020-04-14

**Authors:** Iordana A. Shopova, Ivan Belyaev, Prasad Dasari, Susanne Jahreis, Maria C. Stroe, Zoltán Cseresnyés, Ann-Kathrin Zimmermann, Anna Medyukhina, Carl-Magnus Svensson, Thomas Krüger, Viktòria Szeifert, Sandor Nietzsche, Theresia Conrad, Matthew G. Blango, Olaf Kniemeyer, Marie von Lilienfeld-Toal, Peter F. Zipfel, Erzsébet Ligeti, Marc Thilo Figge, Axel A. Brakhage

**Affiliations:** a Institute of Microbiology, Friedrich Schiller University, Jena, Germany; b Department of Molecular and Applied Microbiology, Leibniz Institute for Natural Product Research and Infection Biology (HKI), Jena, Germany; c Research Group Applied Systems Biology, Leibniz Institute for Natural Product Research and Infection Biology (HKI), Jena, Germany; d Department of Infection Biology, Leibniz Institute for Natural Product Research and Infection Biology (HKI), Jena, Germany; e Clinic of Internal Medicine II, Haematology and Oncology, Jena University Hospital, Jena, Germany; f Department of Physiology, Semmelweis University, Budapest, Hungary; g Centre for Electron Microscopy, Jena University Hospital, Jena, Germany; h Research Group Systems Biology and Bioinformatics, Leibniz Institute for Natural Product Research and Infection Biology (HKI), Jena, Germany; i Friedrich Schiller University, Jena, Germany; Universidad de Córdoba

**Keywords:** *Aspergillus fumigatus*, azurocidin, cathepsin G, extracellular vesicle, fluorescent image analysis, fungi, microvesicle, neutrophils, polymorphonuclear leukocytes

## Abstract

Polymorphonuclear granulocytes (PMNs) are indispensable for controlling life-threatening fungal infections. In addition to various effector mechanisms, PMNs also produce extracellular vesicles (EVs). Their contribution to antifungal defense has remained unexplored. We reveal that the clinically important human-pathogenic fungus Aspergillus fumigatus triggers PMNs to release a distinct set of antifungal EVs (afEVs). Proteome analyses indicated that afEVs are enriched in antimicrobial proteins. The cargo and the release kinetics of EVs are modulated by the fungal strain confronted. Tracking of afEVs indicated that they associated with fungal cells and even entered fungal hyphae, resulting in alterations in the morphology of the fungal cell wall and dose-dependent antifungal effects. To assess as a proof of concept whether the antimicrobial proteins found in afEVs might contribute to growth inhibition of hyphae when present in the fungal cytoplasm, two human proteins enriched in afEVs, cathepsin G and azurocidin, were heterologously expressed in fungal hyphae. This led to reduced fungal growth relative to that of a control strain producing the human retinol binding protein 7. In conclusion, extracellular vesicles produced by neutrophils in response to A. fumigatus infection are able to associate with the fungus, limit growth, and elicit cell damage by delivering antifungal cargo. This finding offers an intriguing, previously overlooked mechanism of antifungal defense against A. fumigatus.

## Abstract (precis)

Invasive fungal infections caused by the mold Aspergillus fumigatus are a growing concern in the clinic due to the increasing use of immunosuppressive therapies and increasing antifungal drug resistance. These infections result in high rates of mortality, as treatment and diagnostic options remain limited. In healthy individuals, neutrophilic granulocytes are critical for elimination of A. fumigatus from the host; however, the exact extracellular mechanism of neutrophil-mediated antifungal activity remains unresolved. Here, we present a mode of antifungal defense employed by human neutrophils against A. fumigatus not previously described. We found that extracellular vesicles produced by neutrophils in response to A. fumigatus infection are able to associate with the fungus, limit growth, and elicit cell damage by delivering antifungal cargo. In the end, antifungal extracellular vesicle biology provides a significant step forward in our understanding of A. fumigatus host pathogenesis and opens up novel diagnostic and therapeutic possibilities.

## INTRODUCTION

The clinical management of invasive aspergillosis, a severe systemic infection mainly caused by the ubiquitous saprophytic fungus Aspergillus fumigatus, is a challenging endeavor. Invasive aspergillosis is characterized by high mortality rates related to the difficult diagnosis, the occurrence of resistance to antifungals, and the lack of novel antifungal therapies (1–6). Invasive aspergillosis can occur in patients with congenital or therapy-induced myeloid cell defects, whereas healthy individuals that continuously inhale fungal spores (conidia; 2 to 3 μm) usually remain symptom free. Data from neutropenic mice and patients have shown that polymorphonuclear granulocytes (PMNs) are indispensable for antifungal defense (7–16); however, the exact mechanism of PMN-dependent fungal killing remains unresolved.

PMNs orchestrate immune surveillance against pathogenic fungi via oxidative burst (14, 17, 18), degranulation (19, 20), phagocytosis (21), cytokine release (7), and extracellular trap formation (11, 16, 22, 23). Neutrophil extracellular traps are only slightly fungistatic, and this alone does not explain the full antifungal activity of PMNs (22, 23). In addition to these effector mechanisms, PMNs also produce PMN-derived extracellular vesicles (EVs), which represent extracellular phosphatidylserine-containing microparticles (50 nm to 1 μm) that elicit pleiotropic immunomodulatory effects in recipient host cells (24–28). PMN-derived EVs serve many functions *in vivo* (29–32), including antibacterial (33–35) and antiviral (36) defense, and have been used as diagnostic markers for sepsis (37). Previous work also indicated that opsonization of bacteria is required for the production of antibacterial PMN-derived EVs (34).

In this report, we demonstrate the immune functionality of PMN-derived EVs against the important filamentous fungal pathogen A. fumigatus. We phenotypically characterized the EVs produced by PMNs in response to A. fumigatus infection and further detail the properties, locations, and antifungal effects of these EVs on the fungus.

## RESULTS

### PMNs release EVs in response to A. fumigatus infection.

**FIG 1:**
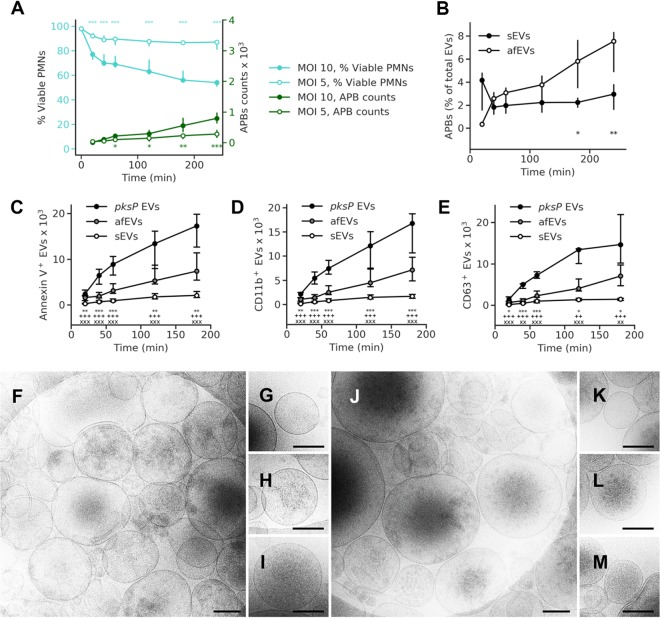
A. fumigatus induces EV release by human neutrophils. (A) Time course of apoptotic body (APB) occurrence (green lines) and fungus-induced cell death (teal lines) at MOIs of 5 and 10 (*n* = 10 [15] and *n* = 12 [17] for apoptotic body counts for MOIs of 5 and 10, respectively; *n* = 4 [20] and *n* = 5 [15] for viability data for MOIs of 5 and MOI 10, respectively) (numbers in brackets are total number of technical replicates). (B) Percentage of apoptotic bodies per total number of EVs. (C to E) Time course of total EV release and the levels of the EV surface markers annexin V (*n* = 27 [40] for sEVs, *n* = 16 for afEVs and *pksP* EVs) (C), CD11b (*n* = 23 for sEVs, *n* = 16 for afEVs and *pksP* EVs) (D), and CD63 (*n* = 13 for sEVs, *n* = 9 for afEVs and *pksP* EVs) (E). sEVs were collected from uninfected cells. Symbols represent significant differences between *pksP* EVs and afEVs (*), *pksP* EVs and sEVs (+), afEVs and sEVs (x). The data in panels A and B to E are presented as the medians and interquartile ranges of the absolute numbers of EVs per 10^7^ PMNs. *P* values were determined by the Mann-Whitney test. *, *P* < 0.05; **, ++, and xx, *P* < 0.01; *** and xxx, *P* < 0.001. (F to M) Cryo-TEM images of sEVs (F to I) and afEVs (J to M) at 2 h postinteraction. Representative images display sEVs (G to I) and afEVs (K to M) with different appearances. Bars, 200 nm.

The confrontation of PMNs with A. fumigatus conidia is known to result in the rapid internalization of the fungus and the production of reactive oxygen intermediates and neutrophil extracellular traps over time (22, 38). In response to opsonized bacterial pathogens, neutrophils have been shown to release antibacterial EVs (34, 39), yet the role of EVs in antifungal defense in mammals remains unexplored. As such, we enriched and characterized PMN-derived EVs produced from viable human PMNs (>95% purity, >98% viability) during infection with opsonized wild-type (wt) A. fumigatus conidia (see Fig. S1A in the supplemental material). To limit PMN apoptosis and the subsequent production of apoptotic bodies, we first determined the apoptotic fate of PMNs over the course of interaction with A. fumigatus by monitoring propidium iodide (PI) and annexin V staining of cells using flow cytometry (Fig. 1A). Both EVs and apoptotic cells expose on the outer leaflet of the cell membrane phosphatidylserine, which can be detected by annexin V. However, in contrast to apoptotic bodies, EVs remain intact and thus impermeable to PI (24–28). By size discrimination using flow cytometry, we could also distinguish between cellular apoptosis and the release of apoptotic bodies (annexin V-positive and PI-positive [PI^+^] EVs) (Fig. 1A and Fig. S1B to E). Coincubation of human PMNs with fungi for 4 h at a multiplicity of infection (MOI) of 5 conidia to 1 PMN triggered minimal cell death in the PMN population (<10%) and limited apoptotic body release compared to an MOI of 10 (Fig. 1A). An MOI of 5 was thus used throughout the remainder of the study to phenotypically characterize PMN-derived EVs.

10.1128/mBio.00596-20.1 FIG S1 Flow cytometry analysis of PMN-derived EVs. (A) Purity of human PMNs used to generate PMN-derived EVs. Purity was assessed by flow cytometry based on staining for CD66b^+^ CD16b^+^ PMNs, CCR3^+^ eosinophils, CD14^+^ monocytes, CD3^+^ T cells, CD19^+^ B cells, CD56^+^ NK cells, and NKT cells. The predominant contaminating fraction of cells was eosinophils. (B to C) Analysis of apoptotic bodies defined as double-positive (annexin V- and PI-positive) EVs. PMNs cultured for 3 days on Dulbecco modified Eagle medium at 37°C were used as a positive control for apoptosis (left). The results for two donors are shown in the center and right panels. (D and E) Flow cytometry protocol for phenotyping afEVs. (D) The instrumental background noise by pure HBSS was recorded and subsequently subtracted from the values for all samples. (E) The results for midintensity rainbow beads of 3.8 μm were recorded to set the upper detection limit; afEVs are detected in the gate above the noise and below the beads. (F and G) Single-stained afEVs with the corresponding isotype antibodies were used as negative controls. Stained afEV suspensions were measured before (F) and after (G) detergent treatment with 1% (vol/vol) Triton X-100 to verify the vesicular nature of the detected events. False-positive events (detergent resistant) were subtracted from the results. Download FIG S1, TIF file, 0.1 MB. Copyright © 2020 Shopova et al. 2020 Shopova et al. This content is distributed under the terms of the Creative Commons Attribution 4.0 International license.

We were particularly interested in the phosphatidylserine-containing and PI-negative fraction of EVs, which was previously linked to host immunity and which can be interrogated by flow cytometry (24–28). Labeling of EVs with cell surface markers for the α-chain of the integrin receptor CR3 (CD11b) and the tetraspanin CD63 revealed an increase in the size of the populations of EVs produced in response to infection with wt A. fumigatus (antifungal EVs [afEVs]) relative to the size of the populations of spontaneously released EVs (sEVs) from uninfected cells (Fig. 1B to E; Fig. S1F and G and S2A and B). When we compared the afEV formation induced by stimulation of PMNs with wt and *pksP* mutant conidia, which lack the pigment and the virulence determinant dihydroxynaphthalene melanin (25, 40–43), we discovered that melanin-deficient conidia doubled the production of EVs analyzed here (Fig. 1C to E). This finding suggests that fewer EVs are produced against melanized wt conidia, consistent with a known repressive role for dihydroxynaphthalene melanin against the host immune response during infection (44). For clarity, we have defined EVs induced by wt conidia as antifungal EVs (afEVs), EVs induced by *pksP* mutant conidia as *pksP* EVs, and spontaneously produced EVs as sEVs. Despite this major difference in EV production, PMN viability was similar for wt and *pksP* mutant conidium-infected cells (Fig. S2C); however, *pksP* conidia did exhibit higher opsonization (Fig. S2D) (42). The vesicular nature of the detected EVs was verified by detergent treatment using 1% (vol/vol) Triton X-100, which led to the disappearance of the signals for both annexin V and EV surface marker staining (Fig. S1F and G). Cryo-transmission electron microscopy (cryo-TEM) imaging (Fig. 1F to M) confirmed a heterogeneous population of circular structures with lipid bilayers for both afEVs and sEVs (26, 45). Both types of EVs appeared to contain cargo with different spatial organizations (Fig. 1G to I and K to M), including practically empty EVs (Fig. 1G and K), granular structures (Fig. 1H and L), and a homogeneous distribution of cargo (Fig. 1I and M). The meaning of the different grade of granularity awaits further attention.

10.1128/mBio.00596-20.2 FIG S2 Characterization of afEV surface markers by flow cytometry. (A) Flow cytometry measurement of PMN surface marker dynamics of CD11b and CD63 during infection with wt and *pksP* conidia at an MOI of 5. PMNs were gated according to forward scatter/side scatter properties, dead cells were excluded by staining with viability Zombie dye, and the expression of CD11b and CD63 was analyzed with FlowJo software (TreeStar). (B) Size distribution of afEVs, *pksP* EVs, and sEVs generated at different time points, as measured by dynamic light scattering. Data are representative of those from 3 independent experiments. (C) Time course of apoptotic body occurrence (green lines) compared to that of fungus-induced cell death (teal lines) for wt and *pksP* infected PMNs. Data are represented as the medians and interquartile ranges. Data for EVs are shown as absolute or relative vesicle numbers per 10^7^ PMNs. *P* values were determined by the Mann-Whitney test. *, *P* < 0.05; **, *P* < 0.01; ***, *P* < 0.001. (D) Opsonization of wt and *pksP* mutant conidia as determined by flow cytometry for C3 immunofluorescence staining. Bars indicate the mean fluorescence intensity plus standard deviation from 2 experiments with 5 replicates each. Download FIG S2, TIF file, 0.1 MB. Copyright © 2020 Shopova et al. 2020 Shopova et al. This content is distributed under the terms of the Creative Commons Attribution 4.0 International license.

### afEVs are enriched for antimicrobial proteins.

**FIG 2:**
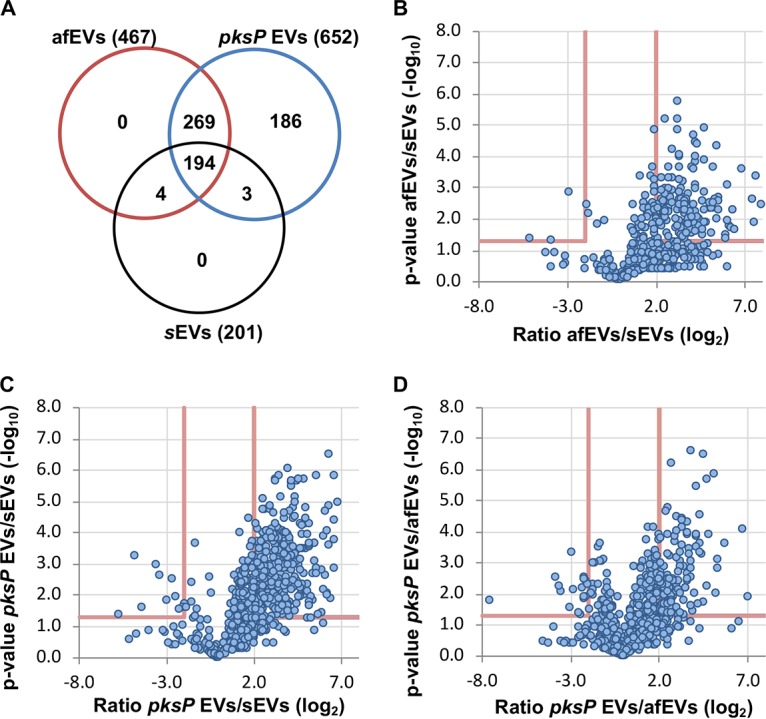
Analysis of the EV proteome by LC-MS/MS reveals that neutrophil-derived EVs retain a core proteome cargo after infection. (A) Venn diagram (created with Venny [version 2.1.0] software) indicating the overlap of proteins identified from each EV population using label-free quantification. (B to D) Volcano plots comparing proteins identified in afEVs, *pksP* EVs, and sEVs using the LFQ-based proteomics method.

We next addressed the cargo of EVs in response to infection. We purified proteins from afEVs, *pksP* EVs, and sEVs from about 10 liters of fresh human blood. Equal amounts of protein were labeled with tandem mass tags (TMT) or left unlabeled for a subsequent label-free quantification (LFQ), followed by detection with nanoscale liquid chromatography (nLC)-tandem mass spectrometry (MS/MS); see the two data sets at https://doi.org/10.6084/m9.figshare.11973174). LFQ analysis revealed an expanded proteome in the afEVs and *pksP* EVs compared to the sEVs, which is suggestive of additional functionality (Fig. 2A). We next compared (i) *pksP* EVs and afEVs, (ii) afEVs and sEVs, and (iii) *pksP* EVs and sEVs. We observed that the afEVs and *pksP* EVs were, again, quite different from the sEVs, but even the afEVs differed from the *pksP* EVs (Fig. 2B to D). Analysis using the TMT method of quantification also indicated differences in each population, consistent with the LFQ data (Fig. S3A to C). Since EVs are often enriched for membrane proteins, we next predicted transmembrane domain-containing proteins using three different tools (TMHMM [46], SignalP [47], and WoLF PSORT [48]) and identified 17 proteins in the TMT data set and 29 proteins in the LFQ data set (Table S1).

10.1128/mBio.00596-20.3 FIG S3 Neutrophil EV composition differs depending on the stimuli. (A to C) Volcano plots comparing proteins identified in afEVs, *pksP* EVs, and sEVs using the TMT-labeling proteomics method. (D) Gene Ontology (GO)-term enrichment analysis of the core proteome cargo (60 proteins), based on the FungiFun2 tool, reveals the pathways of EV biogenesis. The data are representative of those from 2 technical replicates. Download FIG S3, TIF file, 0.3 MB. Copyright © 2020 Shopova et al. 2020 Shopova et al. This content is distributed under the terms of the Creative Commons Attribution 4.0 International license.

10.1128/mBio.00596-20.8 TABLE S1 Identified proteins with transmembrane domains predicted by use of the SignalP, TMHMM, and WoLF PSORT tools based on the TMT and LFQ data sets obtained here. Download Table S1, PDF file, 0.1 MB. Copyright © 2020 Shopova et al. 2020 Shopova et al. This content is distributed under the terms of the Creative Commons Attribution 4.0 International license.

In comparison to sEVs, both afEVs and *pksP* EVs contained a broader spectrum of proteins and, more importantly, larger amounts of antimicrobial peptides, such as neutrophil elastase (NE), myeloperoxidase (MPO), cathepsin G, azurocidin, and defensin 1 (Table 1). CD11b and CD63 were enriched in afEVs and *pksP* EVs compared to sEVs, thus confirming the flow cytometry data (Fig. 1D and E; see the two data sets at https://doi.org/10.6084/m9.figshare.11973174). In addition, afEVs contained larger amounts of metabolic enzymes, such as glucose-6-phosphate isomerase and transketolase, the cell surface glycoprotein CD177, and F-actin. Proteins of the antimicrobial calprotectin complex (S100-A8, S100-A9) exhibited the highest absolute abundance in afEVs (see the two data sets at https://doi.org/10.6084/m9.figshare.11973174). Finally, afEVs and *pksP* EVs were more similar in protein content in comparison to that in sEVs (Table 1; see the two data sets at https://doi.org/10.6084/m9.figshare.11973174).

**TABLE 1:** Selected examples of differentially produced proteins with known antimicrobial activity

UniProt accession no.	Protein	TMT ratio		
		*pksP* EVs/afEVs	afEVs/sEVs	*pksP* EVs/sEVs
A0A0U1RRH7	Histone H2A	1.32	14.08	18.55
U3KQK0	Histone H2B	1.12	6.27	7.04
P68431	Histone H3.1	0.82	16.84	13.79
P08246	Neutrophil elastase	0.62	4.64	2.86
P05164	Myeloperoxidase	1.19	2.16	2.57
P08311	Cathepsin G	0.73	3.07	2.24
P20160	Azurocidin	0.70	3.46	2.41
P59665	Defensin 1	2.04	13.41	27.37

The comparison of the proteins from all EV subsets revealed that 60 proteins were shared between all groups, suggesting that these proteins are part of the core EV protein set. Gene Ontology (GO)-term enrichment analysis of the 60 shared proteins revealed the overrepresentation of proteins involved in Fcγ receptor signaling, Arp2/3 complex-mediated actin nucleation, the interleukin-8 signaling pathway, cytoskeletal rearrangements, and the positive regulation of actin polymerization (Fig. 2; Fig. S3D). In comparison to the findings described in the literature, we found 164 proteins in common between the study of Timar et al. (34) and this study. We detected 118 proteins unique to the study of Timar et al. (34) and 448 proteins unique to our study using LFQ-based proteomics. Infection with wt or *pksP* conidia led to the formation of afEVs and *pksP* EVs with distinct proteome cargos, characterized by increased levels of antimicrobial peptides and metabolic proteins. These findings suggest an antimicrobial function for afEVs.

### afEVs influence fungal growth by inhibition of hyphal extension.

**FIG 3:**
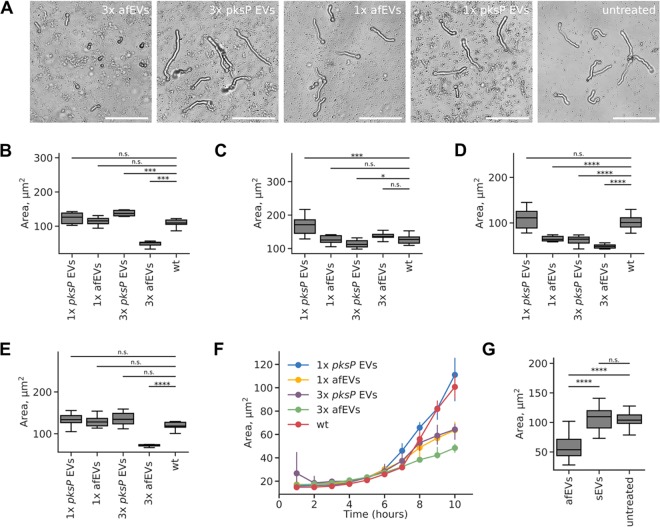
afEVs elicit antifungal effects on wt fungus. (A) Representative bright-field images after 10 h of incubation of wt fungal hyphae with afEVs and *pksP* EVs. Single (1×) or triple (3×) doses of EVs were applied. (B to E) Growth of wt fungal hyphae after 10 h of coincubation with afEVs and *pksP* EVs derived from four different donors. The size of the hyphae was assessed by automated analysis of 2D image data, and the results are displayed as the median hyphal area in each field of view; data are represented as medians and interquartile range of the median hyphal area in each field of view (*n* = 10 fields of view per condition per time point). (F) Representative growth curves of the wt fungal strain in the presence and the absence of EVs for the donor for which the results are shown in panel D. (G) Effects of sEVs on wt conidia compared to those of afEVs on wt conidia (*n* = 3 independent experiments, 20 fields of view per experiment per condition). *P* values were determined by the Mann-Whitney test. n.s., not significant; *, *P* < 0.05; ***, *P* < 0.001; ****, *P* < 0.0001.

To prove a potential antifungal activity of afEVs, we collected afEVs and *pksP* EVs from PMNs, coincubated them in different concentrations with resting conidia, and monitored fungal growth by confocal laser scanning microscopy (CLSM) (Fig. 3A and Fig. S4A and B). The area of objects (single hyphae or clusters) was considered the growth measure. The concentration of EVs was measured in “doses” and is more fully described in Materials and Methods. One dose of EVs was defined as the number of *pksP* EVs produced by 10^7^ PMNs infected with *pksP* mutant conidia at 2 h postinfection. At this time point, we found a relatively large amount of produced EVs (Fig. 1C) associated with a relatively low fraction of apoptotic bodies (Fig. 1B). The doses for each condition were normalized according to abundance from the observations in Fig. 1C. The afEVs generated by PMNs infected with wt conidia strongly inhibited the growth of wt and *pksP* hyphae in all donors when higher doses of EVs were applied (Fig. 3B to E and Fig. S4C to F). These experiments revealed donor heterogeneity in response to four different blood donors, suggesting that the antifungal potential of EVs may differ between individuals. Higher (triple) doses of *pksP* EVs, as well as lower (single) doses of afEVs, were efficient in the growth arrest of hyphal filaments for one donor only (Fig. 3D; Fig. S4E). The antifungal effects of afEVs for all donors were not due to the delayed germination of conidia but, rather, resulted from the inhibition of hyphal extension (Fig. 3A and F; Fig. S4A, B, and G). Interestingly, sEVs had no impact on the growth of fungi (Fig. 3G). Thus, PMNs produce tailored afEVs with distinct functional properties in response to coincubation with A. fumigatus.

10.1128/mBio.00596-20.4 FIG S4 Effect of afEVs on *pksP* mutant fungal cells. (A) Segmentation steps of an automated algorithm for 2D image analysis of fungal growth with (top rows) and without (bottom rows) afEVs. Bars, 20 μm. (B) Representative bright-field images after 10 h of incubation of fungi with afEVs and *pksP* EVs on *pksP* mutant hyphae. Untreated hyphae received no EVs. Single (1×) or triple (3×) doses of EVs were applied as described in Materials and Methods. (C to F) Growth of *pksP* mutant fungal hyphae after 10 h of coincubation with afEVs and *pksP* EVs derived from four different donors. The size of the hyphae was assessed by automated analysis of 2D image data, and the results are displayed as the median hyphal area (in square micrometers) in each field of view; data are represented as the medians and interquartile ranges of the median hyphal area in each field of view (*n* = 10 fields of view per condition per time point). (G) Representative growth curves of the *pksP* mutant fungal strain in the presence and absence of EVs for the donor for which the results are shown in panel E. Download FIG S4, TIF file, 0.2 MB. Copyright © 2020 Shopova et al. 2020 Shopova et al. This content is distributed under the terms of the Creative Commons Attribution 4.0 International license.

### afEVs associate with fungal cells.

**FIG 4:**
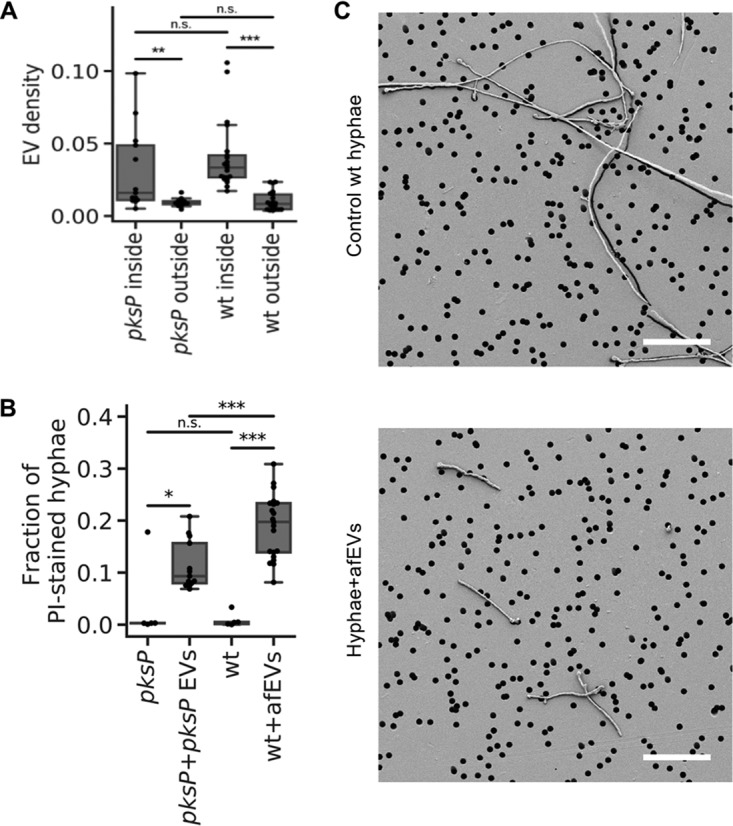
Effect of afEVs on hyphae. (A) Density of afEVs and *pksP* EVs inside and outside of wt and *pksP* mutant hyphae. (B) The fraction of PI-stained hyphae indicates permeable fungal hyphae and provides an estimation of the hypha-associated DNA signals in wt and *pksP* hyphae treated with afEVs and *pksP* EVs, respectively, compared to those in untreated control hyphae. The data in panels A and B for the EV groups were derived from 3 independent experiments (*n* = 13 and 21 technical replicates for *pksP* and wt, respectively). The data in panel B for the controls are representative of those from 1 experiment (*n* = 5 technical replicates). *P* values were determined by the Mann-Whitney test. n.s., not significant; *, *P* < 0.05; **, *P* < 0.01; ***, *P* < 0.001. (C) SEM images of 50-h-old cultures of wt hyphae treated with afEVs (bottom) versus healthy hyphae grown alone (top). Samples were immobilized on filter membranes with a defined pore size of 5 μm (black circles). Bars, 50 μm. SEM images represent observations from 2 independent experiments with 3 technical replicates.

As discussed above, we observed that afEVs are capable of arresting fungal growth. To study the interactions of afEVs with fungi, we collected three-dimensional (3D) confocal fluorescent image stacks of wt hyphae coincubated with afEVs and *pskP* hyphae coincubated with *pksP* EVs after 20 h of incubation. We quantified the interactions of EVs and hyphae using 3D image analysis to evaluate the densities of EVs within (inside) calcofluor white-stained hyphae (in which the EV volume inside the hyphae was normalized to the hyphal volume) compared to the corresponding EV densities outside the hyphal cell wall determined by staining. The densities of EVs inside the hyphae (indicating an association with or internalization of the EVs) were significantly higher than the EV densities outside the hyphae (in which EVs were unassociated with the hyphae) for both wt and *pksP* hyphal filaments (Fig. 4A; see the first two movies at https://doi.org/10.6084/m9.figshare.11973174). The 3D image analysis of the fluorescence signals revealed the extensive binding of EVs induced by conidia of both fungal strains to hyphae, despite the interrogation of equal volumes of EVs and hyphae (Fig. S5A and B).

10.1128/mBio.00596-20.5 FIG S5 Testing of hyphae and EV volume. (A, B) Equal volumes of EVs (A) and hyphae (B) for wt and *pskP* samples were analyzed. (C) Dependence of the volume fraction of the hypha-associated afEVs (volume of hypha-associated afEVs divided by the total afEV volume) on the volume concentration of afEVs (total afEV volume divided by the sample volume). (D) Dependence of the volume fraction of hypha-associated EVs on the volume concentration of hyphae (hyphal volume divided by the sample volume). (E) Dependence of the volume fraction of the hypha-associated DNA (HADNA) that interacts with EVs on the volume fraction of hypha-associated EVs. Download FIG S5, TIF file, 0.1 MB. Copyright © 2020 Shopova et al. 2020 Shopova et al. This content is distributed under the terms of the Creative Commons Attribution 4.0 International license.

We further assessed the ability of afEVs to associate with hyphae by evaluating the volume of hypha-associated EVs, which were defined as the sum of the volumes of afEVs bound to the cell wall or internalized into hyphae (see the first two movies at https://doi.org/10.6084/m9.figshare.11973174). The ability of afEVs to associate with hyphae was mainly dependent on the intrinsic properties of the donors’ afEVs (Fig. S5C), while the relative volume density of afEVs had a much smaller effect (Fig. S5D and E). We next defined hypha-associated DNA staining as PI^+^ signals colocalized with hyphae, which is indicative of hyphal cell damage. The amount of hypha-associated DNA staining from hyphae incubated with afEVs was significantly larger than the amount of hypha-associated DNA staining from control hyphae grown alone, as quantified by the hypha-associated DNA staining-positive volume normalized to the hyphal volume (Fig. 4B; see the second movie at https://doi.org/10.6084/m9.figshare.11973174). The 3D image analysis also showed that PI^+^ staining of hyphae was associated with the interaction of hypha-associated EVs. In fact, more than 60% of the volume of PI^+^ hyphae was associated with hypha-associated EVs (Fig. S5E; see the second movie at https://doi.org/10.6084/m9.figshare.11973174). All donor EVs were capable of eliciting PI staining of hyphae, but the extent of this effect was donor dependent (Fig. S5E). Our data imply that afEVs are antifungal and appear to cause cell damage in a process likely associated with the physical interaction of hyphae and afEVs. In support of this finding, hyphae also appear to undergo hyperbranching away from the afEV layer in response to treatment (see the third movie at https://doi.org/10.6084/m9.figshare.11973174), again suggesting antifungal activity.

The effect of afEVs on fungi led us to test for physical long-term alterations of cell wall morphology. To visualize these changes, we obtained scanning electron microscopy (SEM) images of wt hyphae at 50 h after afEV treatment. The treated hyphal filaments (Fig. 4C) were again shorter, further confirming the antifungal nature of afEVs. Additional imaging showed slight alterations in the porousness of the cell surface, which included ruffling and invaginations that were not observed in hyphae grown without afEVs (Fig. S6).

10.1128/mBio.00596-20.6 FIG S6 SEM imaging of afEV-treated hyphae. SEM images of 50-h-old cultures of wt hyphae treated with afEVs (bottom) versus healthy hyphae grown alone (top). Samples were immobilized on filter membranes with a defined pore size of 5 μm (as seen in the overview images). Bars, 1 μm (images on the far right) and 5 μm (all other images). SEM images represent observations from 3 technical replicates from 2 independent experiments. Download FIG S6, TIF file, 0.2 MB. Copyright © 2020 Shopova et al. 2020 Shopova et al. This content is distributed under the terms of the Creative Commons Attribution 4.0 International license.

**FIG 5:**
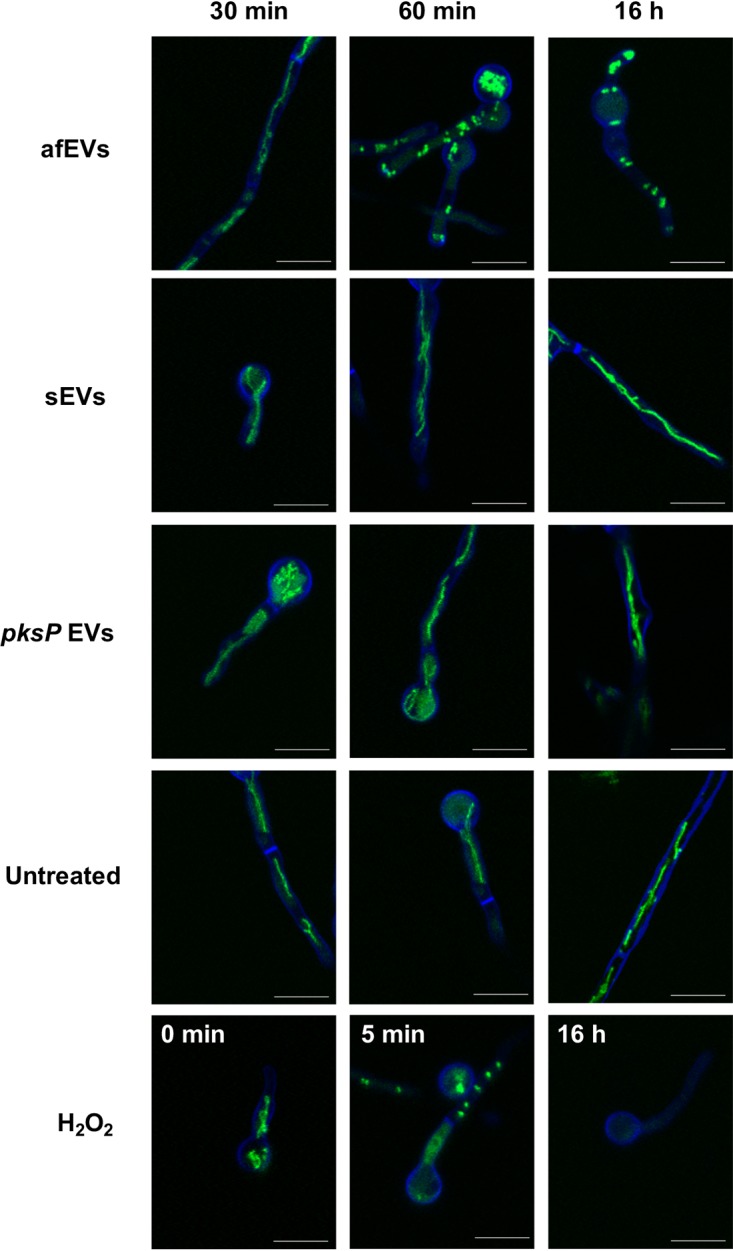
afEVs kill fungal hyphae. AfS35/pJW103 hyphae expressing a mitochondrial GFP reporter (green) grown for 20 h were stained with calcofluor white (blue) and incubated with sEVs, afEVs, *pksP* EVs, or 3 mM H_2_O_2_ as a positive control for cell death induction or left untreated and then monitored by CLSM. A healthy filamentous mitochondrial network is shown in green in an untreated sample. A fragmented mitochondrial network indicates cell death, as seen when 3 mM H_2_O_2_ was used as a positive control for cell death. Images are representative of those from 4 separate experiments with samples from different donors. Bars, 10 μm.

Next, we took advantage of a previously reported mitochondrial green fluorescent protein (GFP) cell death reporter strain (AfS35/pJW103) produced to monitor the granulocyte killing of A. fumigatus (49). In this strain, a mitochondrion-localized GFP indicates filamentous, healthy mitochondria in living fungi, but the mitochondria become fragmented upon initiation of cell death pathways and ultimately lose their fluorescence at later time points. Using this strain, we were able to observe mitochondrial fragmentation and the limited growth of 20-h-old hyphae challenged with afEVs or an H_2_O_2_ control (3 mM) but not of those challenged with *pksP* EVs or sEVs (Fig. 5). These results are consistent with a potential fungicidal activity for afEVs and agree with the results from Fig. 3.

**FIG 6:**
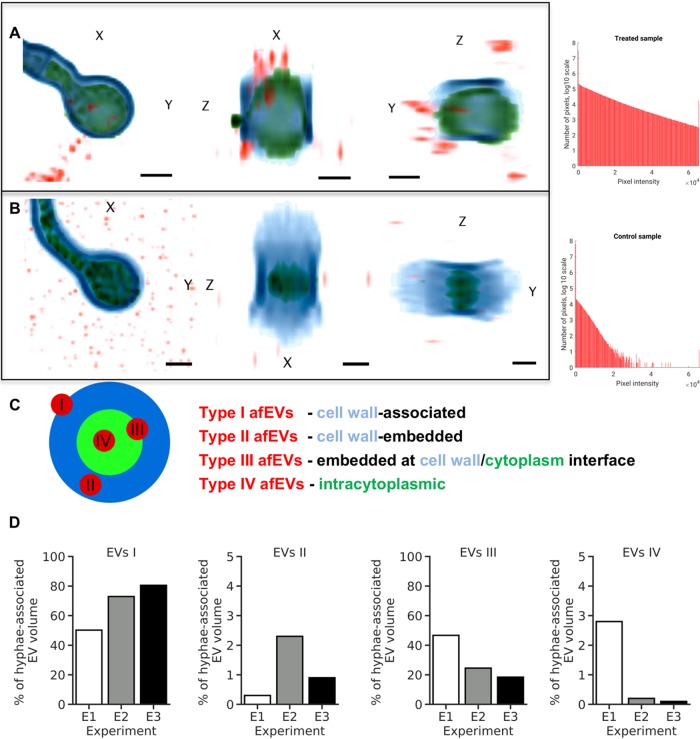
afEVs are internalized into the fungal cell wall and cytoplasm. afEV internalization into fungi was analyzed by 3D quantitative analysis of z-stacks with GFP-expressing A. fumigatus after 20 h of coincubation. (A, B) (Left) Representative cross sections of z-stacks showing lateral (*X* and *Y*) and axial (*Z*) dimensions of a hypha with internalized afEVs (A) and the corresponding control hypha (B). Internalized afEVs are in red (Alexa Fluor 647), the fungal cell wall is in blue (calcofluor white), and the fungal cytoplasm is in green (GFP). The image intensity was inverted. The darkest color corresponds to the highest fluorescence intensity. Bars, 2 μm. (Right) Histograms display the specificity of the signal of the Alexa Fluor 647 dye used to stain afEVs. As seen in the control z-stack, there is unspecific Alexa Fluor 647 staining, likely due to dye aggregation. (C) Schematic diagram of a cross section of hyphae and different stages of afEV internalization. afEVs were located in 4 areas, as indicated by the graphical representation. (D) Overview from the 3D image analysis of different locations of afEVs. Data are representative of those from 3 independent experiments with a total of 25 z-stacks.

To further support our findings of afEVs in association with fungal cells, we performed 3D image analysis of afEV entry into GFP-expressing hyphae. The data obtained demonstrated that afEVs could be incorporated into the fungal cytoplasm (Fig. 6A to D; see the second movie at https://doi.org/10.6084/m9.figshare.11973174). Furthermore, we were able to differentiate four locations of EV-fungal interactions: (i) the largest fraction of afEVs, 50 to 70% (referred to as type I afEVs), were cell wall-associated EVs; (ii) afEVs embedded into the cell wall amounted to 0.5 to 2.5% of the EVs; (iii) 15 to 45% of the afEVs were found to be located at the interface between the cell wall and the cytoplasm; and (iv) intracytoplasmic afEVs represented 0.2 to 3% of all afEVs (Fig. 6A to D; see also the second movie at https://doi.org/10.6084/m9.figshare.11973174).

### afEV proteins are toxic to fungal cells.

**FIG 7:**
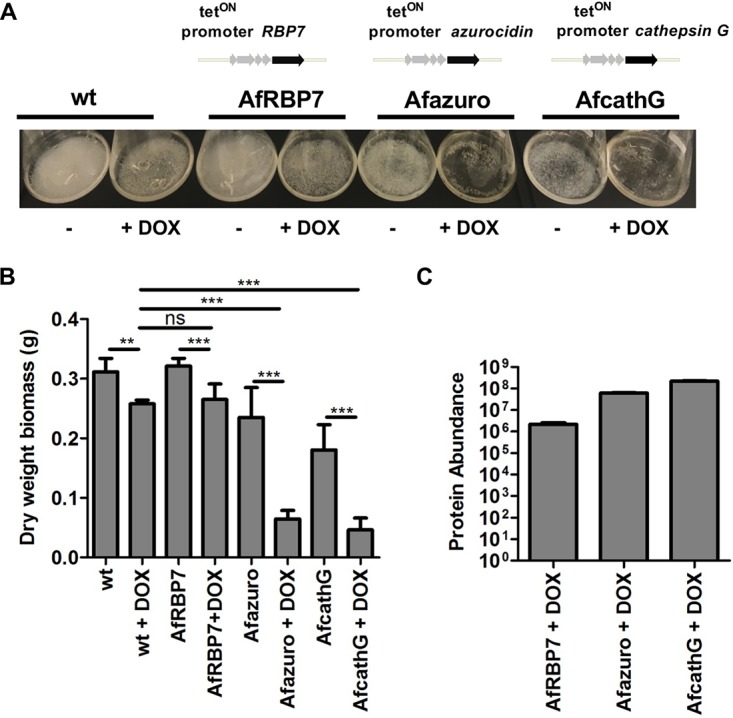
The intracellular production of human azurocidin and cathepsin G proteins is toxic to A. fumigatus. (A) A. fumigatus wt and mutant strains Afazuro, AfcathG, and AfRBP7 harboring the human azurocidin, cathepsin G, and *RBP7* genes, respectively, under the control of the tet^ON^ promoter. The cultures were grown for 24 h in the absence or the presence of doxycycline (DOX). (B) Biomass measurement of wt and A. fumigatus mutant strains Afazuro, AfcathG, and AfRBP7 with and without doxycycline. Data are representative of those from 3 independent experiments with 3 technical replicates. *P* values were determined by the Mann-Whitney test. ns, not significant; **, *P* < 0.01; ***, *P* < 0.001. (C) Detection of proteins produced in the A. fumigatus mutant strains. The bar plot shows the abundance level of the azurocidin protein for the Afazuro strain, the cathepsin G protein for the AfcathG strain, and RBP7 for the AfRBP7 strain, based on the intensity of the precursor ion. The data were generated from 3 analytical replicates.

We next assessed whether the antimicrobial proteins found in afEVs might contribute to the growth inhibition of hyphae when expressed heterologously in the fungus. The genes of two of these human proteins, cathepsin G and azurocidin, were selected because both proteins were enriched in afEVs and are also known to have antifungal effects. For example, cathepsin G knockout mice are highly susceptible to A. fumigatus infection (50, 51). The genes encoding these proteins were placed under inducible expression in A. fumigatus hyphae (Fig. S7A and B). As a control, we also placed the human retinol binding protein 7 (RBP7), a protein detected in EVs with no expected antifungal activity, under inducible expression in A. fumigatus hyphae as well (strain AfRBP7). Addition of the inducer doxycycline to cultures of the transgenic A. fumigatus strains (strains AfcathG and Afazuro) led to a massive growth reduction, whereas the control RBP7 strain (AfRBP7) revealed no change in dry weight (Fig. 7A and B). These findings are consistent with a potential activity of EV cargo proteins in limiting fungal growth when active in the fungus. The presence of the human proteins in hyphae after induction with doxycycline was confirmed by liquid chromatography (LC)-mass spectrometry (MS) measurements of fungal protein extracts (Fig. 7C).

10.1128/mBio.00596-20.7 FIG S7 Verification of A. fumigatus transgenic strains expressing human EV proteins. (A) Schematic of the predicted BamHI restriction sites in the genomes of the mutant strains following integration of the constructs used to express the human genes in the parental strain: the tet^ON^ promoter, the azurocidin-encoding gene, the cathepsin G-encoding gene, the RBP7-encoding gene, the *tef* terminator, and the *ptrA* cassette (pyrithiamine resistance marker). (B) Southern blot for confirmation of construct integration into the A. fumigatus genome. In the transgenic strains, bands with the expected size of 1.7 kb for the Afazuro strain, 1.4 kb for the AfcathG strain, and 1.4 kb for the AfRBP7 strain were observed. No signal was detected for the nontransformed wt strain. Download FIG S7, TIF file, 0.1 MB. Copyright © 2020 Shopova et al. 2020 Shopova et al. This content is distributed under the terms of the Creative Commons Attribution 4.0 International license.

## DISCUSSION

Neutrophils are critical for the elimination of A. fumigatus from the human host (52); however, the exact extracellular mechanisms of how PMNs kill A. fumigatus hyphae are not known (52). A. fumigatus-triggered neutrophil extracellular traps are slightly fungistatic but do not account for the full activity of neutrophils (22, 23). Here, we show that *ex vivo*-applied human EVs triggered by wt conidia (afEVs) inhibit the growth of hyphae and elicit cell damage, adding a new mode of antifungal defense against A. fumigatus. These results are consistent with previous findings from PMN-derived EVs showing antibacterial effects against Staphylococcus aureus (34). We speculate that afEVs are produced primarily as a result of fungus-driven PMN activation, as apoptotic bodies accounted for less than 10% of the total EV population.

afEV production was increased in response to A. fumigatus infection, as confirmed by flow cytometry. EVs increased with kinetics different from those previously reported for anti-S. aureus PMN-derived EVs, where maximum production was observed at 20 min (34). The interaction of PMNs with A. fumigatus conidia resulted in an enrichment of CD63 on afEVs, which was not observed in antibacterial EVs (34) and which is typically found only on EVs smaller than 100 nm. afEVs were also enriched in MPO, NE, and cathepsin G, consistent with their antifungal function. Interestingly and further supporting the importance of the afEV cargo was the finding that cathepsin G, NE, and calprotectin knockout mice are all highly susceptible to infection with A. fumigatus (11, 50). It is possible that these proteins serve an EV-independent role in host defense; however, many of these proteins have been shown to be associated with EVs in our study and previous studies (26, 34).

Our proteomic analysis of EVs indicated that afEVs and *pksP* EVs contained an expanded proteome compared to that in sEVs; however, nearly all proteins from afEVs were found in *pksP* EVs. Despite this overlap, the abundance of these cargo proteins was quite different. These results are consistent with a hypothesis that the abundance of EV cargo proteins dictates the antifungal nature of that EV population. Our results also suggest that the cargo of afEVs is tailored to the pathogen, as wt and *pksP* conidia elicited different responses. It is important to note that the *pksP* mutant utilized in these studies is not a knockout but instead was derived from a UV-mutagenized strain (53). Previous work has indicated that the phenotypes observed with this strain are due to the inactivation of *pksP* and that they could be fully complemented by the wt *pksP* gene (42, 53). Our findings also suggest a novel function for the fungal virulence factor dihydroxynaphthalene melanin (54, 55) in modulating EV biogenesis and protein cargo. Melanized conidia are less opsonized than nonmelanized conidia and, as a consequence, show reduced phagocytosis by neutrophils, which might lead to lower levels of EV production (42). This hypothesis is also supported by the observation that CD11b and CD63 receptors are differentially expressed on the surface of neutrophils during confrontation with *pksP* and wt conidia.

Our results demonstrate that afEVs associate with fungal hyphae, as evidenced by the high proportion of EVs colocalized with the cell wall of the fungus. In addition, since EVs were found intracellularly, inhibition and killing of the fungus might be due to a combination of these adherence and penetration mechanisms. Although we do not know the mechanisms that govern EV uptake, one hypothesis is that the Fcγ receptor found on the surface of EVs directs the EVs to opsonized fungal surfaces to facilitate entry. Once associated, the exact cargo that is required for the observed antifungal activity is also unknown at this time, but we suspect that it is due to a combination of factors. Human primary neutrophils cannot be genetically manipulated, so as a proof of principle, we instead created A. fumigatus strains that produce cathepsin G and azurocidin using an inducible promoter system. The production of these proteins in the fungus clearly led to a massive growth defect, suggesting that delivery of these cargos could contribute to antifungal activity; however, this experiment offers only a proof of principle. In addition, we observed that fungal hyphae move away from afEVs by hyperbranching, suggesting that the fungus actively avoids afEVs. This finding is consistent with other observations of hyperbranching away from neutrophils during infection (56).

Our 3D image analysis and work with a mitochondrial GFP reporter strain illustrated the potential of afEVs to induce fungal cell damage, while they also revealed the afEV association with hyphae and a possible fungicidal capacity. Interestingly, PMN-generated reactive oxygen species (ROS) were recently shown to induce fungal cell death (14), and perhaps there is a connection between ROS-induced fungal cell death and afEV toxicity. More work will be required to fully elucidate the mechanism of fungal killing by afEVs. Our data did show that the intracellular production of antimicrobial peptides could contribute to a severe inhibition of fungal growth. On the other hand, neutrophil EV-associated effector functions are also known to contribute to innate immune pathology. For example, the surface-bound neutrophil elastase of EVs has been shown to cause extracellular matrix destruction and disease in the lungs of patients with chronic obstructive pulmonary disease (57).

In conclusion, our results suggest that human PMNs release afEVs in response to an A. fumigatus infection. These EVs contain a cargo of antimicrobial proteins that inhibit hyphal growth and kill hyphae. We envision that the analysis of EVs produced in bronchoalveolar lavage fluid represents a potentially useful tool for diagnostic and/or prognostic markers of invasive aspergillosis. Although we hypothesize that afEVs serve as an important factor in the control of pathogenesis during A. fumigatus infection, much work remains to be done to completely unveil the function of these important intercellular mediators.

## MATERIALS AND METHODS

### Ethics statement.

This study was approved by the Institutional Review Board of the Jena University Hospital (approval numbers 2395-10/08 and 5074-02/17) in agreement with the Declaration of Helsinki. Informed consent was obtained for study participation. PMNs were isolated from fresh venous blood collected from healthy adult volunteers after obtaining written consent.

### Strains, growth conditions, and fungal biomass determination.

A. fumigatus ATCC 46645, the GFP-expressing strain AfS148 (58), the melanin-free *pksP* mutant (53), and the mitochondrial GFP reporter strain AfS35/pJW103 (49) were maintained on malt extract (Sigma-Aldrich) agar plates supplemented with 2% (wt/vol) agar for 5 days at 37°C. When appropriate, A. fumigatus ATCC 46645 and the overexpression strains A. fumigatus Afazuro, AfcathG, and AfRBP7 were cultivated on *Aspergillus* minimal medium (AMM) for 3 days at 37°C, as described previously (59). All conidia were harvested in sterile deionized water, filtered through 40-μm-pore-size cell strainers (BD Biosciences, Heidelberg, Germany), washed, and resuspended in deionized sterile water. Spore suspensions were counted in a Thoma chamber and stored at 4°C for no longer than 1 week. Freshly harvested spore suspensions were used for each experiment.

For biomass determination, 10^8^ conidia/ml were inoculated in 100 ml AMM, supplemented with 10 μg/ml doxycycline when needed for induction of the tetracycline-controlled transcriptional activation (tet^ON^) promoter, and grown at 37°C at 200 rpm for 24 h. Mycelia were collected, washed, filtered through Miracloth, and dried at 60°C for 3 days before weighing.

### Opsonization of fungi.

Fresh venous blood was drawn from adult male healthy volunteers, aged 20 to 35 years, after they provided informed written consent and used for preparation of normal human serum (NHS). The volunteers had not taken any anti-inflammatory medications for >10 days and had not consumed alcohol for >3 days prior to donation. NHS was obtained by pooling serum prepared from fresh venous blood from seven healthy human donors. The serum was stored at −80°C until use. The conidia were opsonized in 50% (vol/vol) NHS and 50% (vol/vol) Hanks’ balanced salt solution (HBSS) (HyClone, GE Healthcare) for 1 h at 37°C at 500 rpm in duplicate. The conidia were pelleted by centrifugation at 16,000 × *g* at 4°C for 10 min and subsequently washed three times with HBSS prior to confrontation assays with PMNs.

To measure C3 deposition on the conidial surface after opsonization, the conidia were washed three times with Dulbecco’s phosphate-buffered saline (DPBS) and then incubated with a 1:1,000 dilution of polyclonal goat anti-human C3 serum (Comptech) in 3% (wt/vol) bovine serum albumin (BSA) for 1 h at room temperature (RT). This was followed by addition of a 1:400 dilution of Alexa Fluor 647-conjugated donkey anti-goat IgG (Invitrogen) secondary antibody in 3% (wt/vol) BSA for 1 h at RT. The fluorescence of 10,000 conidial cells was measured by flow cytometry (with a BD LSR II flow cytometer), and the median fluorescence intensity of each conidial population was calculated using FlowJo software (Becton, Dickinson, USA).

### PMN isolation.

PMNs were isolated from fresh venous blood from healthy adult volunteers with a purity above 95% and a viability at 98% as previously described in detail (26) with slight modifications, as follows: blood was collected in K_2_EDTA BD Vacutainer tubes (BD Biosciences), and Biocoll separation solution (Biochrom; GE Healthcare) or PolymorphPrep solution (Progen) was used for gradient centrifugation. Neutrophil purity was determined using an antibody cocktail, as follows: CD3-phycoerythin (clone SK7; dilution, 1:50), CD14-V500 (clone M5E2; dilution, 1:200), CD16-allophycocyanin (APC)-Cy7 (clone 3G8; dilution, 1:50), CD19-Alexa Fluor 700 (clone HIB19; dilution, 1:100), CD56-fluorescein isothiocyanate (FITC) (clone NCAM16.2; dilution, 1:100), and CD66b-peridinin chlorophyll protein (PerCP)-Cy5.5 (clone G10F5; dilution, 1:66), obtained from BD Pharmingen, and CCR3-APC (clone 5E8; dilution, 1:40), obtained from BioLegend. The cells (1 × 10^6^) were blocked with 5% (vol/vol) mouse serum and then stained for CCR3 for 10 min at 37°C. Subsequently, an antibody cocktail mix was applied for staining of the remaining antigens from the above-mentioned panel for an additional 30 min at RT. For cell damage assays at each time point, 2 × 10^6^ neutrophils in 200 μl of HBSS were incubated with PI (5 μg) and Alexa Fluor 647-annexin V (5 μl) for 15 min at RT. Then, the cells were centrifuged at 400 × *g* for 5 min and resuspended in 500 μl DPBS. The fluorescence of 10^4^ gated neutrophils was measured by flow cytometry with a BD LSR II flow cytometer (BD Biosciences) and BD FACSDiva software (version 8.0.1; BD Biosciences). The data were analyzed with FlowJo software.

### EV isolation and characterization.

EVs were prepared by following a procedure described by Timar et al. (34) with slight modifications. PMNs at a density of 1 × 10^7^ cells/ml were confronted with opsonized wt A. fumigatus ATCC 46645 or opsonized A. fumigatus *pksP* mutant conidia at an MOI of 10 or 5 in HBSS with Ca^2+^ and Mg^2+^ (HyClone, GE Healthcare) on a linear shaker (100 rpm) at 37°C for 4 h. EVs produced by uninfected PMNs (sEVs) served as a negative control. At the selected incubation time points, PMNs were sedimented for 10 min at 1,000 × *g* at 4°C on 45° fixed-angle rotor (model FA-45-30-11; Eppendorf). The supernatant was filtered by gravity through sterile polyvinylidene difluoride (PVDF) 5.0-μm-pore-size Millex syringe filters (Merck-Millipore). The EV suspensions were stained with a cocktail of fluorescence-conjugated monoclonal antibodies (PerCP-Cy5.5-anti-human CD63 [clone H5C6; BioLegend], RPE-CD11b [Dako], and FITC-annexin V [BioLegend]) for 20 min at RT and centrifuged on a 45° fixed-angle rotor (model FA-45-30-11; Eppendorf) for 20 min at 4°C at 19,500 × *g*. Corresponding single-stained antibody isotype controls were also prepared (PerCP-Cy5.5 mouse IgG1, κ isotype [clone MOPC-21; BioLegend]; mouse IgG1, κ isotype RPE-CD11b [Dako]). After centrifugation, the supernatant was carefully aspirated and EV pellets were resuspended in the original incubation volume in HBSS.

The size distribution of PMN-derived EVs was recorded with a Nanotrac Flex 180° dynamic light scattering system (Microtrac) at 22°C. At least 20 measurements per sample were performed, and the average hydrodynamic radius was calculated with the sphere approximation using FLEX11 software.

Flow cytometry measurements of EVs were conducted on a BD LSR Fortessa flow cytometer using BD FACSDiva software (version 8.0.1) (BD Biosciences), applying an optimized EV flow protocol (60). Briefly, pure HBSS was used to record instrument noise. The upper size limit detection threshold was set by fluorescent rainbow particles with a midrange intensity and a size of 3.0 to 3.4 μm (BioLegend) resuspended in HBSS. Stained EV suspensions were enumerated in the fluorescent gate above the gate of the negative isotype-labeled controls. Once measured, samples were treated with 1% (vol/vol) Triton X-100 to verify the vesicular nature of the detected events. Detergent-resistant events (false positives) were subtracted from the total measured events using FlowJo software (version 10.0.7) from TreeStar.

### Electron microscopy (cryo-TEM and SEM).

For ultrastructural investigations, isolated EVs were imaged by cryo-transmission electron microscopy (cryo-TEM), and the effects of EVs on fungi were studied by scanning electron microscopy (SEM).

For cryo-TEM imaging, sEVs and afEVs collected at the time point of 2 h were freshly prepared using neutrophils from the same male donor and immediately subjected to imaging. Five microliters of purified pelleted EVs in HBSS was applied to carbon-coated copper grids (type R1.2/1.3; Quantifoil Micro Tools GmbH), and the excess liquid was blotted automatically for 2 s from the reverse side of the grid with a strip of filter paper. Subsequently, the samples were rapidly plunged into liquid ethane (cooled to −180°C) in a cryobox (Carl Zeiss NTS GmbH). Excess ethane was removed with a piece of filter paper. The samples were transferred with a cryo-transfer unit (Gatan model 626-DH) into the precooled cryo-TEM (Philips model CM 120), operated at 120 kV, and viewed under low-dose conditions. The images were recorded with a 2k complementary metal oxide semiconductor (CMOS) camera (model F216; TVIPS, Gauting, Germany).

SEM analysis was used to investigate the effect of the afEVs on the growth of A. fumigatus. Therefore, wt conidia were coincubated with the triple dose of PMN-derived EVs for 50 h in HBSS at 37°C in the dark. At the end of the coincubation time, samples were fixed in 2.5% (vol/vol) glutaraldehyde in HBSS on Isopore membrane TMTP filters with a pore size of 5 μm (Merck-Millipore) for 30 min, followed by washing thrice with HBSS buffer (for 10 min each time). Then, the samples were dehydrated in ascending ethanol concentrations (30, 50, 70, 90, and 96% [vol/vol]) for 10 min at each concentration by thoroughly rinsing the membranes and soaking up the liquids with blotting paper. Subsequently, the ethanol was changed to hexamethyldisilazane (Merck) in two steps (50%, 96% [vol/vol]), and the samples were air dried. Afterwards, the samples were sputter coated with gold (thickness, approximately 4 nm) using an SCD005 sputter coater (Bal-Tec, Liechtenstein) to avoid surface charging and investigated with a field emission (FE) SEM LEO-1530 Gemini microscope (Carl Zeiss NTS GmbH).

### LC-MS/MS-based proteome analysis of EVs.

For proteome analysis of EVs, purified sEVs, afEVs, and *pksP* EVs were collected from a pool of 20 different donors in HBSS and stored at −80°C for no longer than 1 week prior to protein extraction. EV suspensions were concentrated on 3-kDa-molecular-mass-cutoff polyethersulfone (PES) membrane centrifugal filters (VWR International) for 5 min at 14,000 rpm at 4°C (Sigma 3-KIS centrifuge). Samples were snap frozen in liquid N_2_ and delipidated by protein precipitation, based on the protocol of Wessel and Flügge (61). Proteins were resolubilized in 50 μl 50 mM triethyl ammonium bicarbonate (TEAB) in 1:1 trifluoroethanol (TFE)-H_2_O and denatured for 10 min at 90°C. Protein quantification was performed using a Direct Detect system (Merck-Millipore). Each sample was set to 40 μg of total protein in 100 μl in 100 mM TEAB. Proteins were reduced with 10 mM Tris(2-carboxyethyl)phosphine (TCEP) at 55°C for 60 min and alkylated with 12.5 mM iodoacetamide (IAA) at RT for 30 min in the dark. Proteins were digested for 2 h at 37°C with C-type lysozyme (Lys-C) and for 16 h at 37°C with trypsin gold (both from Promega). For TMT 6-plex labeling (Thermo Fisher Scientific, Waltham, MA), the digested peptides were treated according to the manufacturer’s instructions. Labeled peptides were pooled and fractionated offline on HyperSep strong-cation-exchange (SCX) columns (Thermo Fisher Scientific).

LC-MS/MS analyses and protein database searches were performed as described by Baldin et al. (62) with the following modifications. Gradient elution using eluent A (0.1% [vol/vol] formic acid in water) and eluent B (0.1% [vol/vol] formic acid in 90:10 acetonitrile-water [vol/vol]) was as follows: 0 to 4 min at 4% eluent B, 15 min at 5.5% eluent B, 30 min at 7% eluent B, 220 min at 12.5% eluent B, 300 min at 17% eluent B, 400 min at 26% eluent B, 450 min at 35% eluent B, 475 min at 42% eluent B, 490 min at 51% eluent B, 500 min at 60% eluent B, 515 to 529 min at 96% eluent B, and 530 to 600 min at 4% eluent B. Precursor ions were measured in full scan mode within a mass range of *m/z* 300 to 1,500 at a resolution of 140,000 full width at half maximum (FWHM) using a maximum injection time of 120 ms and an automatic gain control (AGC) target of 3 × 10^6^ (TMT) or 1 × 10^6^ (LFQ). The isolation width was set to *m/z* 0.8 (TMT) or 2.0 (LFQ) atomic mass units. Tandem mass spectra were searched for by the use of Proteome Discoverer (PD) software (version 2.1; Thermo Fisher Scientific, Waltham, MA) against the UniProt database of Homo sapiens (as of 22 August 2016) using the algorithms of the programs Mascot (version 2.4.1; Matrix Science), Sequest HT, and MS Amanda (63). Dynamic modifications were oxidation of Met (LFQ) and a TMT 6-plex reaction at Tyr (not considered for quantification). Static modifications were the carbamidomethylation of Cys by iodoacetamide (LFQ) and a TMT 6-plex reaction at Lys and the peptide N terminus. The TMT significance threshold for differentially abundant proteins was set to factor of ≥1.5 (up- or downregulation). The data were further manually evaluated based on the average reporter ion count (≥2 for medium confidence, ≥4 for high confidence). Furthermore, the average variability was observed as a function of the differential regulation and the reporter ion count. Label-free quantification was performed by the precursor ions area method of PD software (version 2.1). The mass tolerance was set to 2 ppm, and the signal-to-noise ratio had to be above 3. The abundance values were normalized based on the total peptide amount. The significance threshold for differential protein abundance was set to a factor of ≥2.0 (up- or downregulation).

### Functional annotation of the EV proteome.

The data set of differentially regulated proteins was filtered by the human serum proteome represented by Piper and Katzmann (64) and, in addition, by keratin, epidermal proteins, and complement component 5α, which were not considered for the proteome comparison. The filtering and the overlap analyses were performed in R using the packages provided by Bioconductor software (65). The GO-term enrichment analysis of the overlapping proteins of the TMT data sets was performed using the FungiFun2 tool (66). The results contain categories determined by Fisher’s exact test and Benjamini-Hochberg-corrected *P* values below 0.05.

### Analysis of heterologously expressed human azurocidin and cathepsin G.

Protein preparation, LC-MS/MS analysis, and a database search for the identification of proteins were essentially performed as previously described (62), except for the following changes. The LC gradient elution was as follows: 0 min at 4% eluent B, 5 min at 5% eluent B, 30 min at 8% eluent B, 60 min at 12% eluent B, 100 min at 20% eluent B, 120 min at 25% eluent B, 140 min at 35% eluent B, 150 min at 45% eluent B, 160 min at 60% eluent B, 170 to 175 min at 96% eluent B, and 175.1 to 200 min at 4% eluent B. Mass spectrometry analysis was performed on a QExactive HF instrument (Thermo Fisher Scientific) at a resolution of 120,000 FWHM for MS1 scans and 15,000 FWHM for MS2 scans. Tandem mass spectra were searched against the UniProt database (7 August 2018; https://www.uniprot.org/proteomes/UP000002530) of Neosartorya fumigata (Af293) and the human protein sequences of azurocidin, cathepsin G, and RBP7, using Proteome Discoverer (PD) software (version 2.2; Thermo Fisher Scientific) and the algorithms of Sequest HT (a version of PD software [version 2.2]) and MS Amanda (version 2.0) software. Modifications were defined as dynamic Met oxidation and protein N-terminal acetylation as well as static Cys carbamidomethylation.

### Determination of EV effects on fungi by CLSM.

For determining the effects of EVs on fungi, EVs were dosed according to cell equivalents. One EV dose was defined as the number of EVs produced by 10^7^ PMNs infected with *pksP* mutant conidia at an MOI of 5 at 2 h postinfection, which represented the maximal observed production of EVs (Fig. 1C) and which corresponded to approximately 10^9^ EVs/ml by nanoparticle tracking analysis with a Malvern NS300 instrument (camera setting, 14; detection threshold, 4). At this time point, *pksP* conidia stimulated double the amount of EVs as wt conidia and 12-fold more than sEVs from the same number of cells. Consequently, the doses were adjusted to appropriately compare equal numbers of EVs. Freshly prepared and portioned EVs were coincubated with 30 μl of a suspension of 10^6^ conidia/ml in HBSS in 12-well chambers (Ibidi GmbH). A confocal laser scanning microscopy (CLSM) system (Zeiss LSM 780; Carl Zeiss SAS) was employed; see “CLSM setup” below for details. Images were acquired once per hour from 10 different fields of view per well in a microtiter plate. The two-dimensional (2D) confocal images were recorded at a pixel size of 208 by 208 nm, whereas 3D image stacks had a voxel volume of 0.025 (19 samples) or 0.034 (15 samples) μm^3^.

After 20 h, the samples were stained with annexin V-FITC (dilution, 1:60; BioLegend), PI (to a final concentration of 0.0167 μg/μl), and calcofluor white (to a final concentration of 0.167 μg/μl) in order to assess EV entry into hyphae and to collect image z-stacks by CLSM. When the GFP-expressing A. fumigatus strain AfS148 was used, the staining cocktail consisted of annexin V-Alexa Fluor 647 and calcofluor white, whereas PI staining was omitted in order to avoid spectral overlaps.

For investigation of EV-mediated fungal killing, we took advantage of a previously described mitochondrial GFP-expressing reporter strain, AfS35/pJW103 (49). When growing normally, this fungal strain shows a normal filamentous network of mitochondria, indicated by mitochondrion-specific fluorescence. For these experiments, 10^6^ conidia/ml of strain AfS35/pJW103 were grown in HBSS in 8-well chambers (Ibidi GmbH) for 20 h prior to coincubation with freshly prepared EV fractions. Here, we used EVs collected from equal amounts of PMNs (10^7^ PMNs). A CLSM system (Zeiss LSM 780; Carl Zeiss SAS) was used to monitor mitochondrial fragmentation (GFP signal) and cell growth (calcofluor white) over time. As a control, cell death was initiated using 3 mM H_2_O_2_, which causes the mitochondria to undergo fusion and form punctate structures within 1 h and then fade in fluorescence signal over time (49).

### CLSM setup.

The imaging data were collected with a Zeiss LSM 780 confocal laser scanning microscope (Carl Zeiss SAS). Images were taken using either a 10× (numerical aperture [NA], 0.4) or a 20× (NA, 0.7) objective lens in an inverted configuration, resulting in a total magnification of ×100 or ×200, respectively. In order to measure the point-spread function of the CLSM system, 5 μl of the blue, green, and deep-red calibration beads from a PS-Speck microscope point source kit (diameter, 170 nm; Invitrogen) was resuspended in the staining cocktail. The bead mixture was imaged under the same conditions applied for the z-stacks of the hypha-EV system. Individual 3D bead images were averaged per color by the use of the HuygensPro program, and the resulting 3D bead images were used to distill the measured point-spread function for all three colors. For the imaging of hyphae and EVs, the CLSM objective lens and stage were preheated to 37°C for 3 to 5 h prior to image scanning. Bright-field images were acquired from 10 different fields of view per well once per hour for 15 time points using a 20× (NA, 0.8) dry objective at 37°C in 5% (vol/vol) CO_2_ atmosphere.

For the z-stacks, images were collected at an axial separation that was set according to the Nyquist criterion for the shortest wavelength, using the same z-step size for all channels. The axial range was adjusted to the thickness of the observed cells.

### 3D image analysis of EV internalization.

For a quantitative analysis of the afEV-hypha interactions, the 3D shape of each object type was reconstructed based on four-dimensional (3D plus color) fluorescence images using the following procedure: the images were deconvolved using the HuygensPro program (Scientific Volume Imaging, Hilversum, The Netherlands) with a measured point spread function (PSF) (see “CLSM setup” above) that was recorded individually for each fluorescence channel. The deconvolved images were transferred to Imaris software (Bitplane, Zürich, Switzerland) for 3D reconstruction. The basic object types (hyphae, DNA, EVs) were reconstructed in Imaris software using manually adjusted templates. The reconstructed hyphae included objects only from the calcofluor white channel (see “Determination of EV effects on fungi by CLSM” above) that were larger than 20 μm^3^ and that had no surface points on the sample border. The reconstruction process is presented in the first and second movies at https://doi.org/10.6084/m9.figshare.11973174. The control samples and those with GFP fluorescence were reconstructed using the same procedure. Hypha-associated DNA and hypha-associated EVs were identified by using a binary mask of the hyphae (channel 4; see the second movie at https://doi.org/10.6084/m9.figshare.11973174). Only those objects that were located either on the border or inside the hyphae, as identified by a threshold of the mean value of the calcofluor white fluorescence signal above 5 × 10^−10^, were considered hypha associated. The binary mask of hypha-associated EVs was used to select hypha-associated DNA that interacted with EVs (hypha-associated DNA, mean value for the binary mask of hyphae; see the second movie at https://doi.org/10.6084/m9.figshare.11973174). Finally, the total volume of each object class at every field of view was computed. Additionally, the EV volume inside the hyphae was computed over the regions that were double positive for annexin V (EVs) and calcofluor white (hyphae), whereas the EV volume outside the hyphae was defined as the volume that was positive for annexin V but not calcofluor white. The EV densities inside and outside the hyphae were then defined as follows: EV density inside hyphae = (EV volume inside hyphae/hyphal volume) and EV density outside hyphae = [(EV volume outside hyphae)/(sample volume − hyphal volume)]. The sample volume was estimated based on the voxel size and the number of voxels in each sample (automatically performed by Imaris software).

### Automated 2D image analysis of hyphal growth.

For quantitative analysis of hyphal growth in bright-field microscopy images, the area of the regions of interest (ROI) corresponding to the conidia and the hyphae was computed automatically for each image. The image analysis algorithm was implemented in Matlab (Matlab 2017a; MathWorks). The code is available from the authors upon request. The procedure included (i) binarization of the image data, (ii) binary image enhancement, (iii) selection of the ROI based on morphological filtering, (iv) image postprocessing and filtering, and (v) measurement of the area of the ROI. Two of the original image sections, together with the resulting images after application of the aforementioned steps, are illustrated in Fig. S4A in the supplemental material. All parameters of the algorithm were adjusted to minimize the detection of noise and of out-of-focus objects, and the adjustment was confirmed by visual inspection of the images. The image data were saved in 16-bit CZI format and loaded into Matlab using the bfopen script from the Open Microscopy Environment (https://www.openmicroscopy.org/site/support/bio-formats5.3/developers/matlab-dev.html).

The images were processed in five steps. In step 1, binarization was performed using the function imbinarize from the Matlab ImageProcessing tool box with the following parameters: the adaptive threshold type, a sensitivity factor for adaptive thresholding of 0.45, and a foreground darker than the background (ForegroundPolarity = dark). In step 2, enhancement of the binary image included the following steps: majority filter, which sets a pixel value to 1 if five or more pixels in its 3-by-3 neighborhood have values of 1 and to 0 otherwise; hole filling inside the ROI; and object removal for ROIs with an area of less than 200 pixels, which corresponds to the minimal area of resting conidia. The resulting image is referred to as image *S*. Step 3 was selection of the ROI, in which image *S* was split into two masks, masks *M* and *S′*, based on the object area, where image *M* contained all ROIs with an area of less than 1,000 pixels, which corresponded to resting, swollen, and germinated conidia, as well as parts of vesicle clumps, and image *S′*, which was all remaining large ROIs, which corresponded to hyphae; removal of all ROIs from mask *M* with a solidity value below 0.85, corresponding to vesicle clumps (the resulting mask is referred to as mask *M′*); and combination of masks *M′* and *S′* into one mask, mask *R*, by the logical sum operation of masks *M′* and *S′*. Step 4 was image postprocessing and filtering, consisting of the morphological closing of mask *R* with two line elements (10 pixels long; orientations, 45° and 135°) to connect broken contours and removal of all ROIs for which the 1st percentile of their Feret diameters was less than 17 pixels (the size of resting conidia). Removal of these ROIs removes the remaining vesicle clumps which have regions thinner than 17 pixels. For Feret diameter calculation, the tool box Feret diameter and oriented box was used (David Legland, https://www.mathworks.com/matlabcentral/fileexchange/30402-feret-diameter-and-oriented-box). Step 5 consisted of measurement of the area of the ROI, in which the area of each object was computed using the function regionprops with the parameter FilledArea and the median of the areas of all ROIs in an image was used to characterize fungal coverage in the image.

### Generation of transgenic A. fumigatus strains.

For expression of the human azurocidin gene (*AZU1*), the human cathepsin G gene (*CTSG*), and the human retinol binding protein 7 gene (*RBP7*) in A. fumigatus, a tetracycline-controlled transcriptional activation (tet^ON^) system was used (67). The human azurocidin, cathepsin G, and retinol binding protein 7 cDNA sequences obtained from the NCBI database were codon optimized for A. fumigatus using the GENEius tool (https://www.eurofinsgenomics.eu/en/gene-synthesis-molecular-biology/geneius/) and synthesized together with the *tef* terminator (Eurofins Genomics). Each of the genes was PCR amplified from the corresponding synthetic template using the Phusion Flash high-fidelity PCR master mix (Thermo Fisher Scientific) with the primer pairs Azu_polictail_f and tef_r for azurocidin, cathG_polictail_f and tef_r for cathepsin G, and RBP7_polictail_F and tef_r for RBP7 (Table S2). The *tet^ON^* promoter cassette was amplified from plasmid pSK562 with primers ptetOn_pYES2tail_F and pOliC_R, while the pyrithiamine resistance cassette (*ptrA*) was amplified from plasmid pSK275 with primers ptrA_teftail_F and ptrA_pYES2tail_R. Plasmid pYES2 was used as the backbone vector and amplified with primers pYES2_r and pYES2_f. The *tet^ON^* cassette, each of the three human genes, and the *ptrA* cassette were assembled with the pYES2 backbone using the NEBuilder HiFi DNA assembly master mix (New England Biolabs) according to the manufacturer’s instructions. The resulting 10-kb plasmids were sequenced and subsequently used to transform A. fumigatus ATCC 46645 as previously described (59). Transformants were selected with 0.1-μg/ml pyrithiamine.

10.1128/mBio.00596-20.9 TABLE S2 Primers used in this study. Download Table S2, PDF file, 0.01 MB. Copyright © 2020 Shopova et al. 2020 Shopova et al. This content is distributed under the terms of the Creative Commons Attribution 4.0 International license.

Southern blot analysis to confirm genetic manipulation of the A. fumigatus strains was carried out as described before (68). For Northern blot analysis, 16-h-old precultures were treated with 10-μg/ml doxycycline. Mycelia were harvested at 3 h after the addition of doxycycline. RNA extraction and detection of RNA by Northern blotting were carried out as previously described (68).

### Data availability.

The mass spectrometry proteomics data have been deposited in the ProteomeXchange Consortium via the PRIDE partner repository with the data set identifier PXD005994 (69).
