# Supplementary figures and images for: Human Neutrophils Produce Antifungal Extracellular Vesicles against Aspergillus fumigatus
Source: mBio. 2020 Apr 14;11(2):e00596-20. doi: 10.1128/mBio.00596-20 (PMC7157820; doi:10.1128/mBio.00596-20)

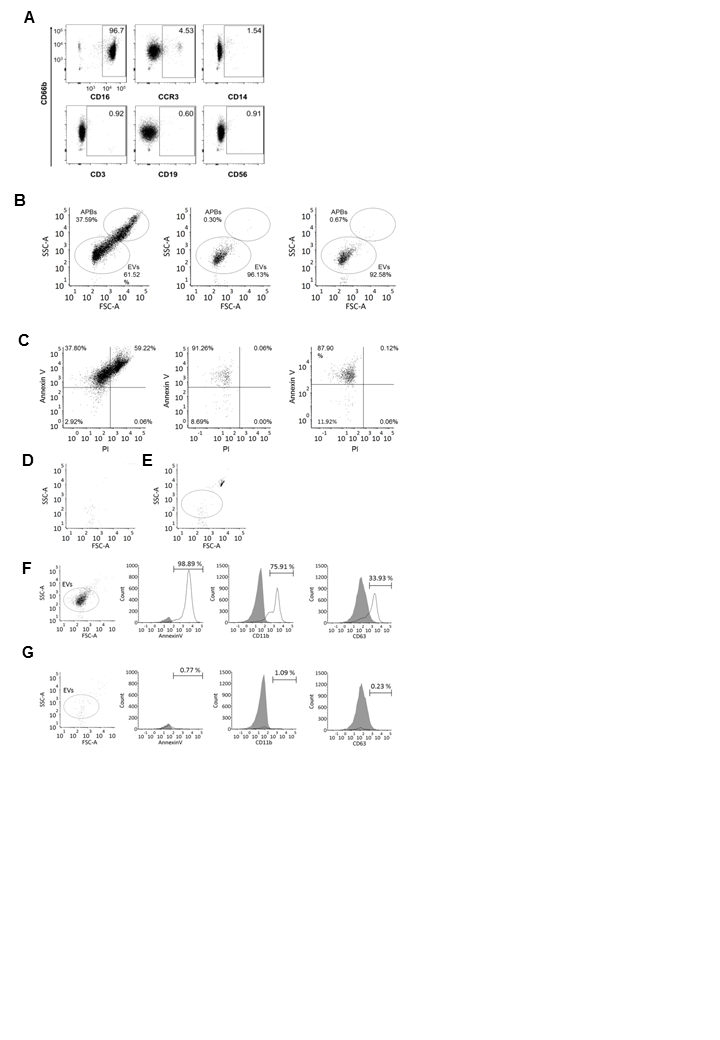

Supplement: FIG S1 [file mBio.00596-20-sf001.tif]

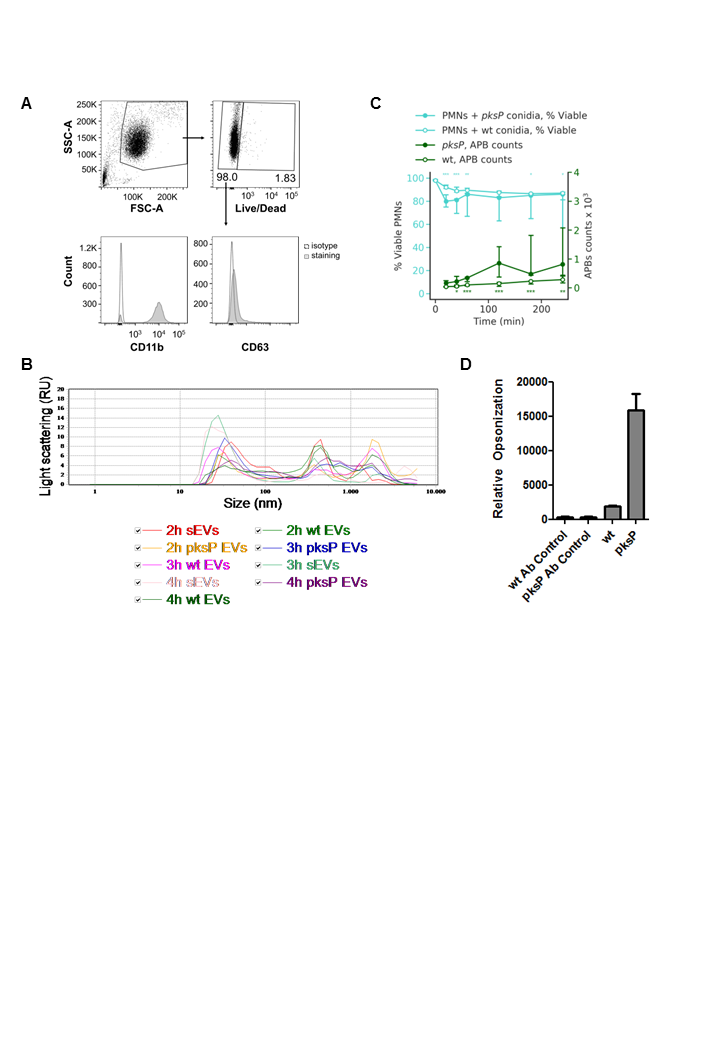

Supplement: FIG S2 [file mBio.00596-20-sf002.tif]

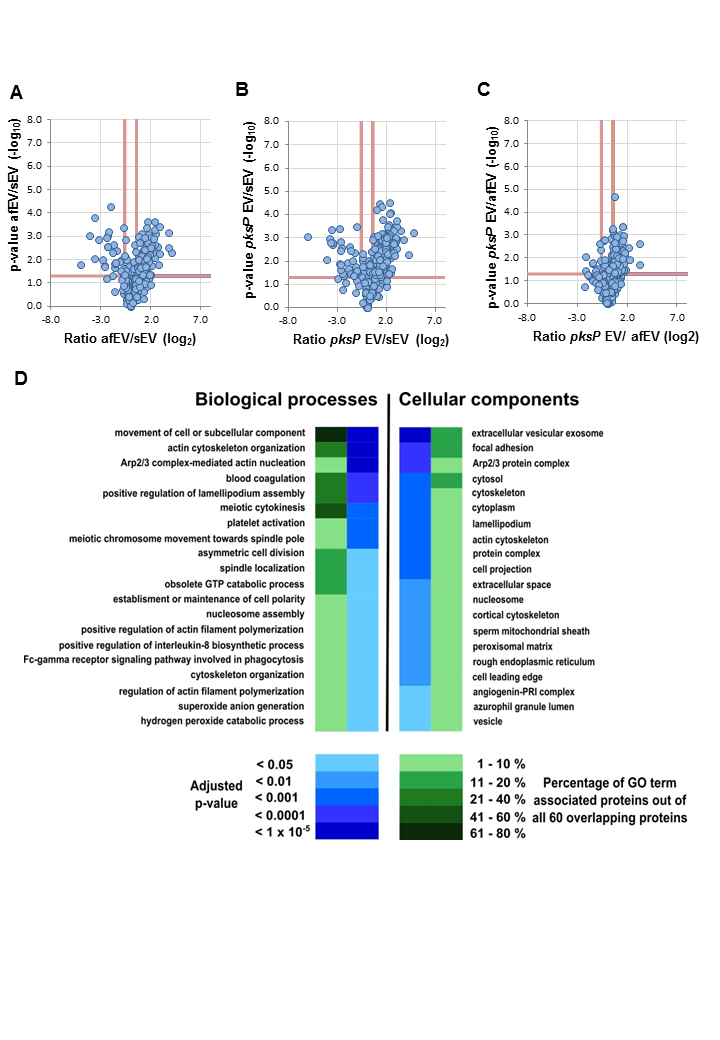

Supplement: FIG S3 [file mBio.00596-20-sf003.tif]

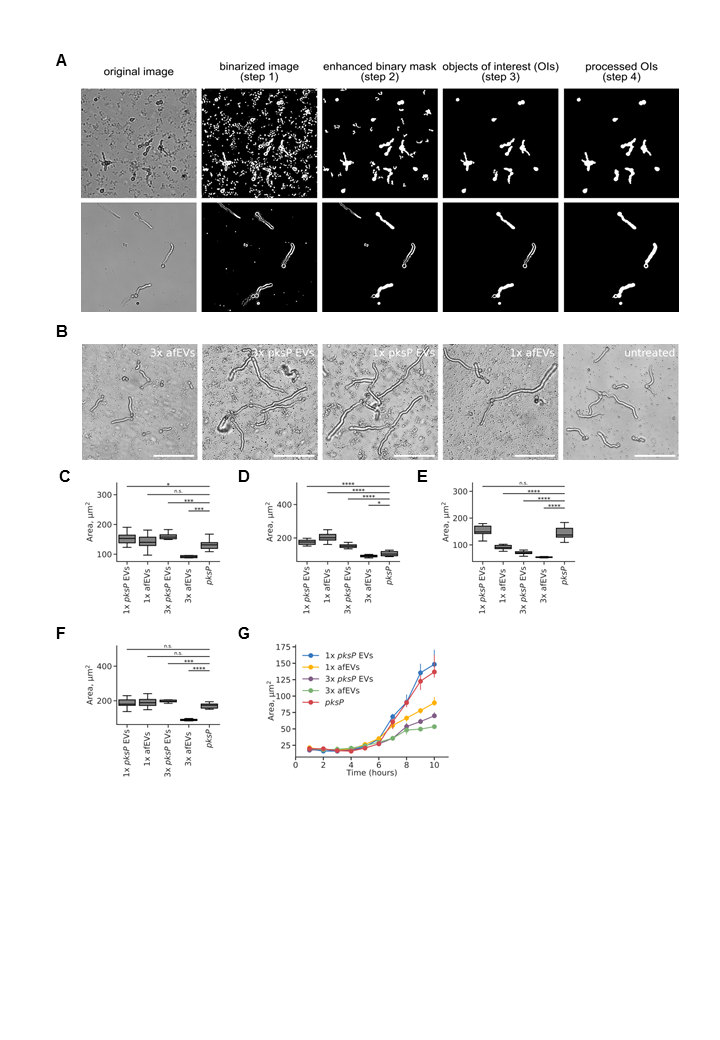

Supplement: FIG S4 [file mBio.00596-20-sf004.tif]

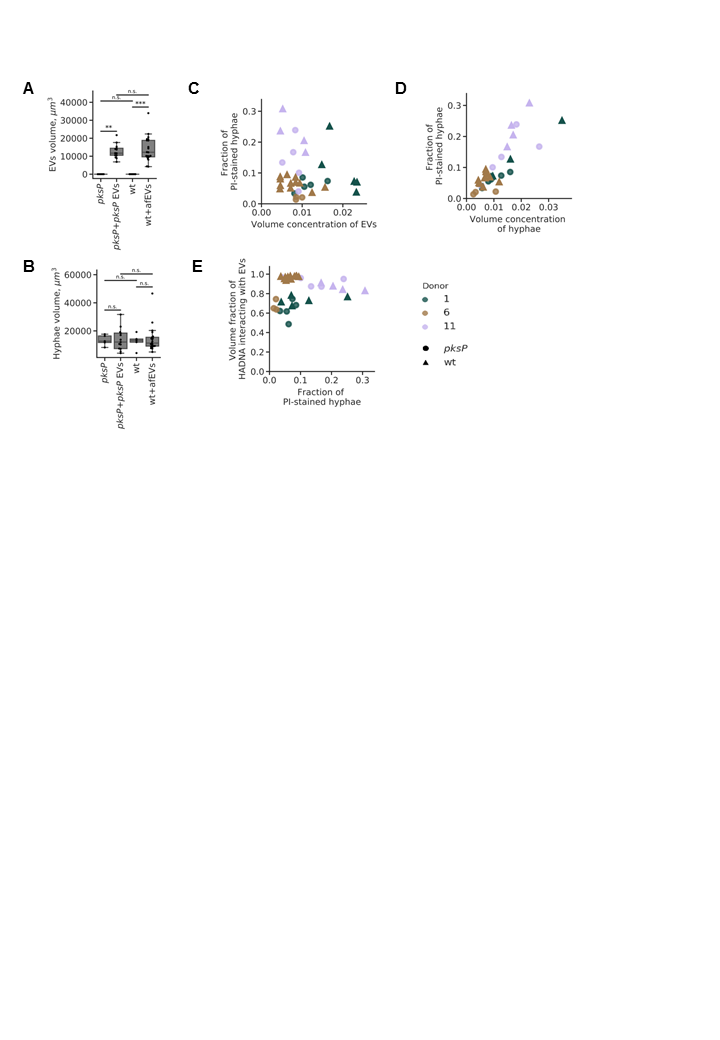

Supplement: FIG S5 [file mBio.00596-20-sf005.tif]

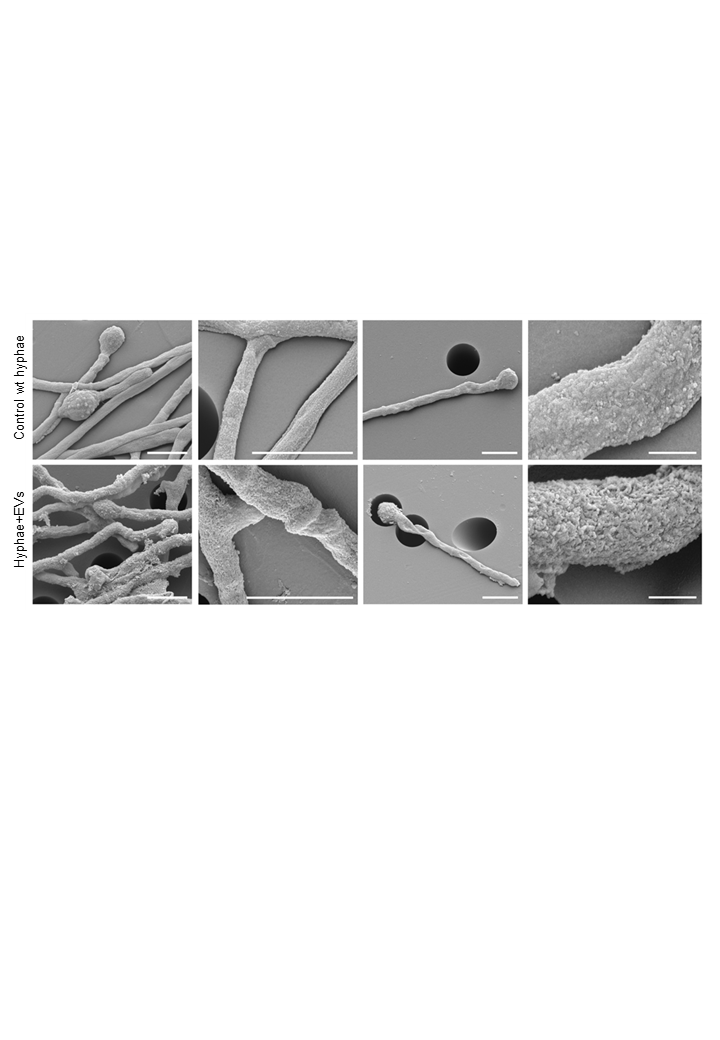

Supplement: FIG S6 [file mBio.00596-20-sf006.tif]

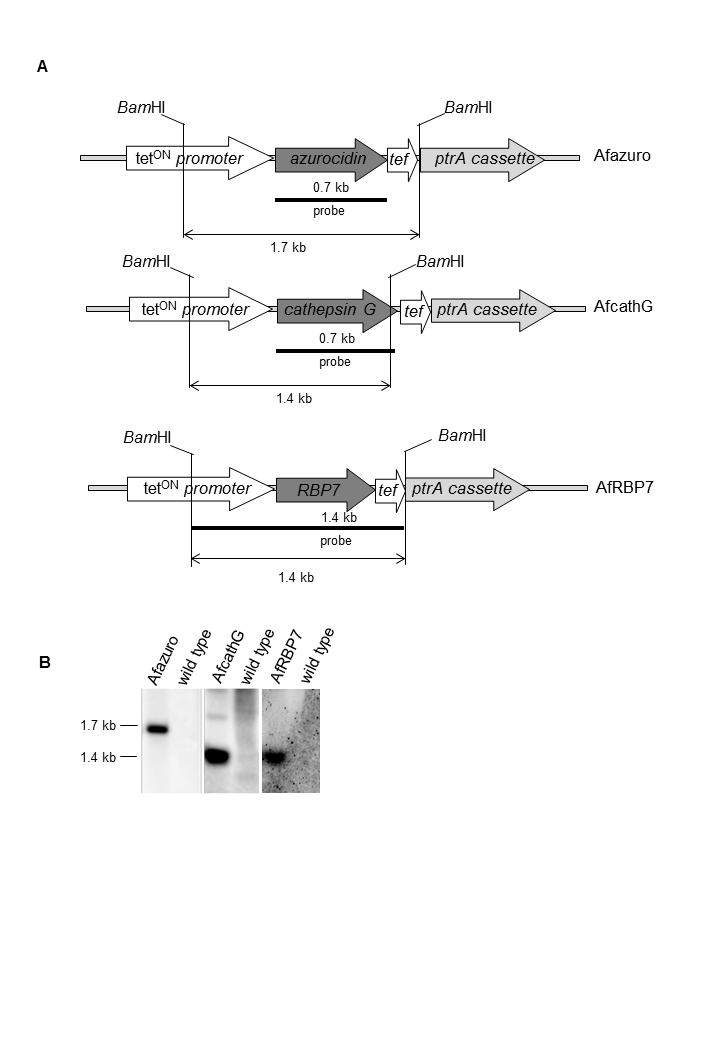

Supplement: FIG S7 [file mBio.00596-20-sf007.tif]
