# Supplementary material for: Human Neutrophils Produce Antifungal Extracellular Vesicles against Aspergillus fumigatus
Source: mBio. 2020 Apr 14;11(2):e00596-20. doi: 10.1128/mBio.00596-20 (PMC7157820; doi:10.1128/mBio.00596-20)
Supplement: TABLE S1 [file mBio.00596-20-st001.pdf]

TABLE S1. Proteins identified with predicted transmembrane domains.

Accession	Protein	TMT dataset	LFQ dataset
P25024	C-X-C chemokine receptor type 1 (CXC-R1)	+	-
P25025	C-X-C chemokine receptor type 2 (CXC-R2)	+	-
Q9HDC9	Adipocyte plasma membrane-associated protein	+	+
P19397	Leukocyte surface antigen CD53	+	-
Q13724	Mannosyl-oligosaccharide glucosidase	+	-
J3KNB4	Cathelicidin antimicrobial peptide	+	-
P15144	Aminopeptidase N	+	+
Q14697	Neutral alpha-glucosidase AB	+	+
P08246	Neutrophil elastase	+	+
M9MML0	Fc of IgG low affinity IIIa receptor isoform 1	+	-
Q8TDB8	Solute carrier family 2, facilitated Glc transporter member 14	+	+
P27105	Erythrocyte band 7 integral membrane protein	+	+
P13498	Cytochrome b-245 light chain	+	+
P08962	CD63 antigen (Granulophysin)	+	-
P04839	Cytochrome b-245 heavy chain	+	-
P17213	Bactericidal permeability-increasing protein (BPI)	+	-
J3KPA1	Cysteine-rich secretory protein 3 CRISP3	+	+
Q53GQ0	Very-long-chain 3-oxoacyl-CoA reductase	-	+
Q96N66	Lysophospholipid acyltransferase 7 (LPLAT 7)	-	+
Q9NV96	Cell cycle control protein 50A	-	+
P08473	Neprilysin	-	+
O43760	Synaptogyrin-2	-	+
P07686	Beta-hexosaminidase subunit beta	-	+
P20701	Integrin alpha-L (CD11 antigen-like family member A)	-	+
F5H2F4	C-1-tetrahydrofolate synthase	-	+
P39656	Dolichyl-diphosphooligosaccharide-protein glycosyltransferase 48 kDa subunit	-	+
P00403	Cytochrome c oxidase subunit 2	-	+
P33121	Long-chain-fatty-acid--CoA ligase 1	-	+
Q15722	Leukotriene B4 receptor 1 (LTB4-R 1)	-	+
I3L0A0	HCG2044781	-	+
Q9NYU2	UDP-glucose:glycoprotein glucosyltransferase 1	-	+
Q8IWA5	Choline transporter-like protein 2	-	+
P13473	Lysosome-associated membrane glycoprotein 2 (LAMP-2)	-	+
Q7L5N7	Lysophosphatidylcholine acyltransferase 2	-	+
O75477	Erlin-1 (Endoplasmic reticulum lipid raft-associated protein 1)	-	+
P21730	C5a anaphylatoxin chemotactic receptor 1	-	+
E7ER45	Maltase-glucoamylase	-	+
P57088	Transmembrane protein 33	-	+
