# Supplementary material for: Human Neutrophils Produce Antifungal Extracellular Vesicles against Aspergillus fumigatus
Source: mBio. 2020 Apr 14;11(2):e00596-20. doi: 10.1128/mBio.00596-20 (PMC7157820; doi:10.1128/mBio.00596-20)
Supplement: TABLE S2 [file mBio.00596-20-st002.pdf]

TABLE S2. List of primers used in this study.

Name	Sequence (5'-3')
Azu_polictail_f	TCACCTCCATCTCAACTCCATCACATCACAATGATCGTCGGCGGCCGCAA
cathG_polictail_f	TCACCTCCATCTCAACTCCATCACATCACAATGATCATCGGCGGCCGCGA
RBP7_polictail_F	TCACCTCCATCTCAACTCCATCACATCACAATGCCCGCAGATCTATCTGG
tef_r	ATTAAGGGTTCTCGAGAGCT
pYES2_r	AATATTCCCTATAGTGAGTC
pYES2_f	CTAGAGGGCCGCATCATGTA
ptetOn_pYES2tail_f	GCTGTAATACGACTCACTATAGGGAATATTTCTTTGCCCGGTGTATGAAA
pOliC_r	CTCAACTCCATCACATCACA
ptrA_teftail_f	GTCGAAAACGAGCTCTCGAGAACCCTTAATCAATTGATTACGGGATCCCA
ptrA_pYES2tail_r	CATAACTAATTACATGATGCGGCCCTCTAGTCTTTCTTGTTACACATAAT
